# Effects of repetitive peripheral magnetic stimulation on upper extremity motor function recovery after stroke: a meta-analysis and dose-response study

**DOI:** 10.3389/fneur.2026.1824669

**Published:** 2026-04-30

**Authors:** Liu Hui, Zhang Lin, Xie Liang, Yang Tianhua

**Affiliations:** 1 Department of Neurology, Yingshan County People's Hospital, Nanchong, China; 2 Department of Neurology, West China Hospital of Sichuan University, Chengdu, China

**Keywords:** dose-response, meta-analysis, motor dysfunction, neuromodulation, repetitive peripheral magnetic stimulation, stroke, upper limb

## Abstract

**Background:**

Repetitive peripheral magnetic stimulation (rPMS), a representative non-invasive neuromodulation technique, is widely utilized for the recovery of motor dysfunction following stroke. Although its clinical efficacy has been confirmed, discrepancies among studies and the optimal rPMS stimulation parameters remain unclear. This study aims to systematically analyze and quantitatively evaluate the optimal stimulation parameters using rPMS parameter subgroups extracted from existing studies.

**Methods:**

Conducted in accordance with PRISMA guidelines, this study searched for research related to rPMS and the Fugl-Meyer Assessment for Upper Extremity (FMA-UE) in stroke patients. A systematic review and meta-analysis of the aggregated studies were then performed. Furthermore, a robust error meta-regression (REMR) model was employed to explore the non-linear dose-response relationship between rPMS stimulation parameters (frequency, intensity, duration, and treatment days) and FMA-UE scores.

**Results:**

A total of 14 trials (*n* = 580) were included. The results indicated that rPMS yielded a significant therapeutic effect on FMA-UE score improvement (SMD = 0.91, 95% CI 0.31–1.51; *p* = 0.003) and spasticity reduction (SMD = −1.15, 95% CI − 1.80 to −0.49; *p* = 0.0006). Dose-response analysis revealed an inverted U-shaped curve for both frequency and duration: the greatest clinical benefits were achieved with optimal stimulation at 10 Hz (peak gain: 13.82 points, 95% CI 9.65–18.00), 10–20 min per session, a plateau effect at 20–55% maximum stimulator output (MSO), and a treatment course of ≥21 days. During the subacute stroke window (14 days to 6 months), neural-targeted stimulation (e.g., brachial plexus, radial nerve) demonstrated superiority over muscle-targeted approaches (SMD = 0.81 vs. 0.47; *p* = 0.006).

**Conclusion:**

Under optimal parameter windows, the therapeutic mechanism of rPMS may be associated with triggering homeostatic plasticity and beta-band corticomuscular coherence. The greatest benefits are obtained particularly with neural-targeted protocols during the subacute phase of stroke, utilizing low frequency (≤20 Hz), moderate-to-low intensity (20–55% MSO), and an extended treatment course (≥21 days). In conclusion, current evidence provides a novel scientific basis and clinical reference for the application of rPMS in stroke rehabilitation.

## 1 Introduction

Currently, post-stroke motor dysfunction represents the leading risk factor for long-term disability, posing a global rehabilitation challenge (1, 2). Research suggests that following a stroke, direct damage to the motor cortex and the corticospinal tract weakens or eliminates the descending drive signals of spinal nerves, which is the primary cause of motor dysfunction (3, 4). This subsequently induces secondary neurological dysfunction, resulting in an inability to inhibit hyperactive reflexes of the spinal neural circuits (e.g., spasticity, increased muscle tone) (5). More importantly, stroke survivors must be guided by rehabilitation professionals during recovery to avoid over-compensation or the exacerbation of motor impairment (6). According to research reports, the annual incidence of stroke currently exceeds 12.9 million and is projected to climb to 89.32 per 100,000 individuals by 2030, a trend particularly pronounced in low-income countries (7). This continuously rising incidence rate, coupled with long-term care costs, will impose a severe economic burden on individuals, families, and society.

To address post-stroke motor dysfunction, active recovery utilizing physical or occupational therapy is universally adopted in clinical practice. Such therapies promote motor function remodeling and compensation through interventions like task-oriented training and constraint-induced movement therapy. However, when the coupling efficiency of neural network plasticity approaches saturation, the efficacy of these therapies often exhibits diminishing marginal returns (8). Furthermore, non-invasive neuromodulation techniques represented by repetitive transcranial magnetic stimulation (rTMS) and transcranial direct current stimulation (tDCS) have been proven to positively impact motor recovery after stroke. Nevertheless, due to their mechanisms of action, these central stimulation techniques are still associated with certain side effects (e.g., headache, nausea, somnolence) (9, 10). Given this, there is an urgent need during the rehabilitation process for an intervention technique capable of activating motor neural networks while avoiding the adverse effects of direct central stimulation. Repetitive peripheral magnetic stimulation (rPMS) acts directly on peripheral targets, thereby avoiding direct stimulation of the central nervous system (11, 12). Meanwhile, rPMS does not rely on the active recruitment of muscle groups by the patient; instead, it passively transmits sensory signals to the central nervous system and stimulates muscle contraction (13). This technical advantage not only significantly enhances patient acceptance and adherence to treatment but also exerts a positive effect on improving post-stroke motor dysfunction and activities of daily living. Although rPMS holds broad prospects, discrepancies in dosage parameters (such as frequency, intensity, and stimulation duration) across studies necessitate a quantitative analysis of the relationship between stimulation parameters and motor outcomes. Therefore, this study aims to utilize the stimulation parameters from various rPMS subgroups to provide a more comprehensive and scientific basis for the clinical application of rPMS.

## 2 Methods

This study has been registered on the PROSPERO platform (registration no: CRD420251245194) and was conducted following the Preferred Reporting Items for Systematic Reviews and Meta-Analyses (PRISMA) guidelines (14).

### 2.1 Literature source and search strategy

Literature retrieval and data collection were conducted according to the Cochrane Handbook standards. A total of four electronic databases (PubMed, Embase, Web of Science, and the Cochrane Library) were searched. The search period was from December 2025 to February 2026. A combination of MeSH terms and free-text words, including “rPMS,” “stroke,” “motor dysfunction,” “upper limb motor dysfunction,” and “repetitive peripheral magnetic stimulation,” along with key text information, was utilized for the search. The specific search strategy can be found in the Supplementary files (see Search Strategy).

### 2.2 Inclusion and exclusion criteria

#### 2.2.1 Inclusion criteria

(1) The study design was restricted to randomized controlled trials (RCTs); (2) Participants (>18 years old) were diagnosed with stroke and presented with upper limb motor dysfunction; (3) The experimental group received any form of rPMS intervention (either alone or combined); (4) The control group received non-rPMS interventions.

#### 2.2.2 Exclusion criteria

(1) Non-RCT study designs (e.g., case reports, conference proceedings, reviews); (2) Non-clinical studies (e.g., animal or cellular models); (3) Missing data precluding analysis; (4) Treatment modalities entirely unrelated to rPMS.

### 2.3 Quality assessment and data extraction

Literature screening was performed independently by two researchers. Initially, irrelevant literature was excluded by screening titles and abstracts, followed by a rigorous full-text review of the remaining potentially relevant articles. A third researcher resolved any discrepancies, and the final included studies were determined through consensus. The primary information extracted included: publication details (author, year), participant characteristics (age, sample size, gender, time since stroke), stimulation parameters (target, frequency, magnetic field intensity, stimulation duration, coil type, treatment course), and interventions (physical therapy, occupational therapy, acupuncture, iTBS).

(1) Motor function outcome measures: Fugl-Meyer Assessment for Upper Extremity (FMA-UE), Action Research Arm Test (ARAT), Wolf Motor Function Test (WMFT), Manual Function Test (MFT), Box and Block Test (BBT), and Fugl-Meyer Assessment for Lower Extremity (FMA-LE). (2) Spasticity assessment measures: Modified Ashworth Scale (MAS), Modified Tardieu Scale (MTS), and elbow flexor bulk-tone-diameter (eBT). (3) Activities of daily living and functional independence: Barthel Index (BI), Functional Independence Measure (FIM), and Modified Barthel Index (MBI). (4) Muscle strength and morphology evaluation: Medical Research Council scale (MRC) and surface electromyography root-mean-square (sEMG RMS). (5) Ultrasound parameters: supraspinatus thickness, deltoid thickness, cross-sectional area (CSA), and supraspinatus tendon thickness (STT). (6) Range of motion and pain assessment: Active Range of Motion (AROM) and visual analogue scale (VAS). (7) Other functional evaluations: Frenchay Arm Test (FAS), American Shoulder and Elbow Surgeons shoulder assessment (AHI), acromion-humeral distance (AHD), acromion-greater tuberosity distance (AGT), and acromion-lateral epicondyle distance (ALT).

The quality of the included studies was evaluated using the Cochrane Risk of Bias (RoB) tool. Specific evaluation criteria included random sequence generation, allocation concealment, blinding of participants and personnel, blinding of outcome assessment, incomplete outcome data, selective reporting, and other sources of bias (15).

### 2.4 Statistical analysis

Effect sizes were analyzed using Review Manager 5.4, and dose-response analyses were performed using Stata 18.0. Since the majority of studies utilized FMA-UE and MAS as core indicators—both being clinical “gold standards” for assessing upper limb motor impairment and muscle spasticity, which objectively reflect the intervention efficacy of rPMS (16), other outcomes were excluded from the main analysis due to limited sample sizes. Given the variations in implementation protocols across studies, data are presented as standardized mean differences (SMD) with 95% confidence intervals (CI). If certain studies did not provide standard deviation values, the mean and standard deviation were estimated using the five-number summary method (17, 18). Following the recommendations of the Cochrane Handbook, the specific effect sizes and statistical differences were calculated using the mean differences and standard deviations between the experimental and control groups post-intervention. The Chi-square test and I^2^ statistic were employed to assess model heterogeneity. A random-effects model was used when heterogeneity was significant (I^2^ ≥ 50%), whereas a fixed-effects model was applied for lower heterogeneity (I^2^ < 50%). Subgroup differences were assessed using the Z-test to calculate the Z and *p* values for the SMD differences between groups. A one-stage robust error meta-regression (REMR) model based on inverse-variance weighted least squares regression and cluster-robust variance was utilized to analyze the dose-response relationship between various rPMS stimulation parameters and FMA-UE (19).

## 3 Results

### 3.1 Literature search results

**Figure 1:**
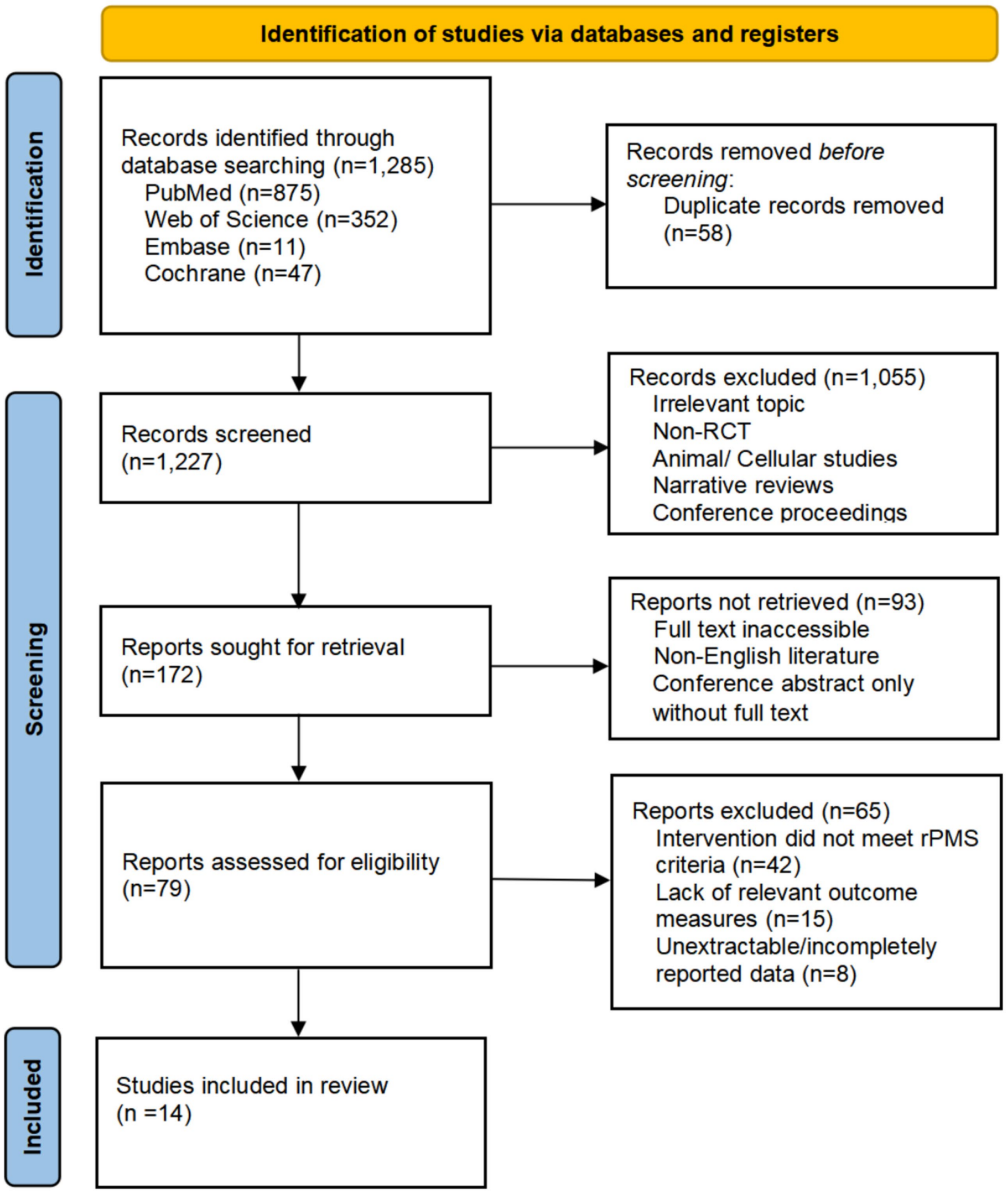
PRIMAS-2020 studies flow diagram.

This study conducted literature screening in strict accordance with the PRISMA 2020 statement. A total of 1,285 relevant records were retrieved from electronic databases, including 875 from PubMed, 352 from Web of Science, 11 from Embase, and 47 from the Cochrane Library. After 58 duplicate records were removed using reference management software, title and abstract screening was performed on the remaining 1,227 records independently by two researchers, with any discrepancies resolved through consensus consultation by a third researcher. A total of 1,055 records were excluded for irrelevant topics, non-randomized controlled trial (non-RCT) design, animal or cellular studies, narrative reviews, or conference proceedings. The remaining 172 records entered the full-text review stage, among which 93 were excluded due to inaccessible full text, non-English literature, or only a conference abstract being available without the complete full text. Eligibility assessment was then conducted on the 79 retained records, and 65 records were excluded in accordance with the predefined inclusion and exclusion criteria: 42 with interventions failing to meet the rPMS criteria, 15 without relevant outcome measures, and 8 with incomplete and unextractable data. Ultimately, a total of 14 eligible RCTs involving 580 participants were included in this study (see Figure 1).

### 3.2 Study characteristics

This study included 14 RCTs published between 2014 and 2024 (20–33), involving 580 post-stroke patients with upper limb motor dysfunction. The male-to-female ratio of participants was approximately 2:1, with an average age ranging from 42 to 72 years. The disease duration spanned from the acute to chronic phases (7 to 1,923 days). Regarding outcome measures, 11 studies utilized FMA-UE as the primary indicator, while MAS analysis was performed on Werner and Mareike (21), Chen et al. (23), El Nahas et al. (27), Aleksandrovich et al. (30), Fawaz et al. (28), and Fujimura et al. (33). All studies targeted the affected upper limb muscle groups, primarily covering key functional muscles such as the extensor digitorum, extensor carpi radialis, flexor digitorum, triceps brachii, biceps brachii, deltoid, and supraspinatus. Among them, 9 studies simultaneously stimulated ≥2 muscles, while 2 studies specifically targeted the brachial plexus (26) and radial nerve (31), respectively. Regarding stimulation parameters, frequencies ranged from 5 to 50 Hz, with 20 Hz (25, 26, 32) and 30 Hz (24, 33) being the most common, though 5 Hz low frequency was also used (21, 22). Stimulation intensity varied significantly (from 10% MCT to 100% RMT), and single-session pulse counts ranged from 690 to 12,000, with figure-eight coils being predominantly applied (*n* = 7). The treatment course varied from 1 to 42 days, administered 5 times daily or weekly, with single session durations lasting 3–40 min. For the control groups, 13 received conventional rehabilitation and 1 received electrical stimulation (22), with all trials combining interventions with physical or occupational therapy. In summary, the significant variance in these stimulation parameters provided the direction for subsequent dose-response analysis (see Table 1).

**Table 1:** Characteristic table of researchers.

Study	Country	Sample size (*n*)	Sex (M/F)	Age (years)	Time since stroke (days)	Target	Frequency (Hz)	Intensity	Pulses	Stimulation/rest (s)	Treatment duration	Coil type	Control intervention	Additional intervention	Outcome measures	Follow-u*p*
Krewer et al. (2014) (20)	Germany	63	38/25 EG: 19/12 CG: 19/13	EG: 53 ± 13CG: 54 ± 13	EG: 182 ± 497CG: 259 ± 574	Upper arm extensors and flexors	25	>10% MCT	5,000	1/2	20 min/session, twice/day for 14 days	Butterfly	Sham (inactive coil)	Occupational therapy	FMA-UE, MTS, BI	14 days
Werner and Mareike (2016) (21)	Germany	40	EG&CG: 16/24	EG&CG: 51.7 ± 10.0	EG&CG: 699 ± 233	Forearm flexors	5	60% MSO	750	3/3	5 min/session, single intervention	Circular	Sham (inactive coil)	Physical therapy	MAS	2 days
Yang et al. (2018) (22)	China	30	23/7 EG: 12/3 CG: 11/4	EG: 63.7 ± 15.1CG: 67.2 ± 10.7	EG: 13.87 ± 3.36CG: 15.47 ± 2.72	Supraspinatus, deltoid	5	100% RMT	NA	NA	40 min/session, 5 times/week for 28 days	Figure-of-eight	Electrical stimulation (FES)	Conventional rehabilitation (45 min/day, 5 times/week) for 28 days	FMA-UE, AGT, ALT, AHD, supraspinatus thickness, deltoid thickness	28 days
Chen et al. (2020) (23)	China	32	23/9 EG: 10/6 CG: 13/3	EG: 49.0 ± 18.2CG: 45.6 ± 8.3	EG: 1122 ± 1260CG: 684 ± 801	Shoulder-elbow-wrist flexors/extensors	20-05-2026	100% MCT	750/5100	3/1, 1.5/1	30 min/session, single intervention	Parabolic	Sham (inactive coil)	NA	FMA-UE, MAS, MTS	1 day
Obayashi and Takahashi (2020) (24)	Japan	19	13/6 EG: 8/2 CG: 5/4	EG: 64.3 ± 13.1CG: 72.3 ± 10.7	EG: 9.2 ± 4.4CG: 5.8 ± 2.2	Extensor digitorum, wrist extensors, finger flexors, triceps brachii, biceps brachii, deltoid	30	70% MSO	NA	2/2	15–20 min/session, 3 times/week until transfer	Circular	Standard care (40 min/day, 5 times/day)	Standard care (20 min/session, 3 times/week)	FMA-UE, WMFT, FAS, BBT	NA
Jiang et al. (2022) (25)	China	44	27/17 EG: 14/10 CG: 13/7	EG: 54.6 ± 10.9CG: 56.1 ± 10.9	EG: 13.8 ± 2.5CG: 14.4 ± 3.3	Triceps brachii, extensor digitorum	20	15–30% MSO	2,400	0.5/2	20 min/session, twice/week for 14 days	Circular	Conventional rehabilitation	Physical therapy	FMA-UE, BI, sEMG RMS	14 days
Ke et al. (2022) (26)	China	26	13/13 EG: 7/6 CG: 7/6	EG: 56.8 ± 4.9CG: 55.3 ± 4.5	EG: 20 ± 10.3CG: 15 ± 5.2	Axilla (brachial plexus), popliteal fossa (tibial/peroneal nerve)	20	40–60% MSO	1800	1/19	30 min/session, daily for 10 days	Figure-of-eight	Sham (coil kept away)	Conventional treatment (30 min PT + 30 min acupuncture/day)	FMA-UE, FMA-LE, MRC	10 days
El Nahas et al. (2022) (27)	Egypt	42	27/9 EG: 20/5 CG: 7/4	EG: 47.9 ± 14.8CG: 41.6 ± 14.9	EG: 128 ± 2.2CG: 192 ± 2.7	Biceps brachii, wrist/finger flexors	50	>MCT	600	2/8	1,600 s/session, once every 2 days for 8 days	Figure-of-eight	Sham	NA	MAS, eBTD	≈14 days
Fawaz et al. (2023) (28)	Egypt	80	EG&CG: 56/24	EG&CG: 57.3 ± 10.67	EG&CG: 42–420	Shoulder abductors, elbow extensors, wrist extensors, supinators	30	>10% MCT	4,500	5/1	30 min/session, 5 times/week for 21 days	Circular + Butterfly	Sham	Occupational therapy (40 min/session)	FMA-UE, FIM, AROM, MAS, ultrasound CSA/STT	21 days
Wu et al. (2023) (29)	China	30	26/4 EG: 11/4 CG: 15/0	EG: 54.9 ± 11.6CG: 55.3 ± 10.3	EG: 30.8 ± 10.6CG: 38.7 ± 11.8	Cervical nerve roots	10	80% RMT	1,000	1/5	1,000 s/session (10 min), 5 times/week for 21 days	Circular + Figure-of-eight	Conventional rehabilitation (60 min/day, 5 times/week)	Physical and occupational therapy	FMA-UE, WMFT, BBI	90 days
Aleksandrovich et al. (2023) (30)	Russia	40	EG&CG: 24/16	EG&CG: 57.3 ± 10.3	NA	Motor points of spastic and antagonist muscles	NA	Individually adjusted	NA	NA	10 sessions	NA	1% stimulation intensity	Conventional physical therapy	MAS, MTS, Arma	NA
Chang et al. (2024) (31)	China	28	14/14 EG: 4/10 CG: 10/4	EG: 51.4 ± 12.1CG: 55.6 ± 10.3	EG&CG: 7- ≥ 180	Radial nerve groove	50	Individually adjusted	1,200	2/8	10 min/session, 5 times/week for 14 days	Figure-of-eight	Sham	PT + OT (60 min + 60 min), iTBS	FMA-UE, ARAT, FIM	14 days
Yan et al. (2024) (32)	China	60	38/22 EG: 18/12 CG: 20/10	EG: 59.3 ± 10.8CG: 58.4 ± 10.4	EG: 59.4 ± 10.7CG: 58.1 ± 10.4	Erb’s point (brachial plexus), deltoid, biceps brachii, brachial nerve	20	Individually adjusted	NA	2/8	3 min/session, 5 times/week for 28 days	Figure-of-eight	Sham (TMS)	Conventional rehabilitation	FMA-UE, VAS pain score, shoulder ROM	28 days
Fujimura et al. (2024) (33)	Japan	46	31/15 EG: 14/8 CG: 17/7	EG: 69.0 ± 13.0CG: 61.0 ± 15.0	EG: 34 ± 23CG: 41 ± 20	Supraspinatus, posterior deltoid/infraspinatus	30	0.65–0.9 Tesla	12,000	2/3	17 min/session, daily for 42 days	NA	Conventional rehabilitation	Conventional rehabilitation (180 min/day)	FMA-UE, AHI	42 days

All values are presented as mean ± SD or *n* (%) unless otherwise specified. EG, experimental group; CG, control group; MCT, maximal contraction threshold; MSO, maximum stimulator output; RMT, resting motor threshold; FMA-UE, Fugl-Meyer Assessment–Upper Extremity; MTS, Modified Tardieu Scale; BI, Barthel Index; MAS, Modified Ashworth Scale; FES, functional electrical stimulation; AGT, acromion-greater tuberosity distance; ALT, acromion-lateral epicondyle distance; AHD, acromion-humeral distance; WMFT, Wolf Motor Function Test; FAS, Frenchay Arm Test; BBT, Box and Block Test; FIM, Functional Independence Measure; AROM, active range of motion; CSA, cross-sectional area; STT, supraspinatus tendon thickness; sEMG RMS, surface electromyography root-mean-square; MRC, Medical Research Council scale; eBTD, elbow flexor bulk-tone-diameter; VAS, visual analogue scale; AHI, American Shoulder and Elbow Surgeons shoulder assessment; PT, physical therapy; OT, occupational therapy; iTBS, intermittent theta-burst stimulation; NA, not available.

### 3.3 Quality assessment and systematic

**Figure 2:**
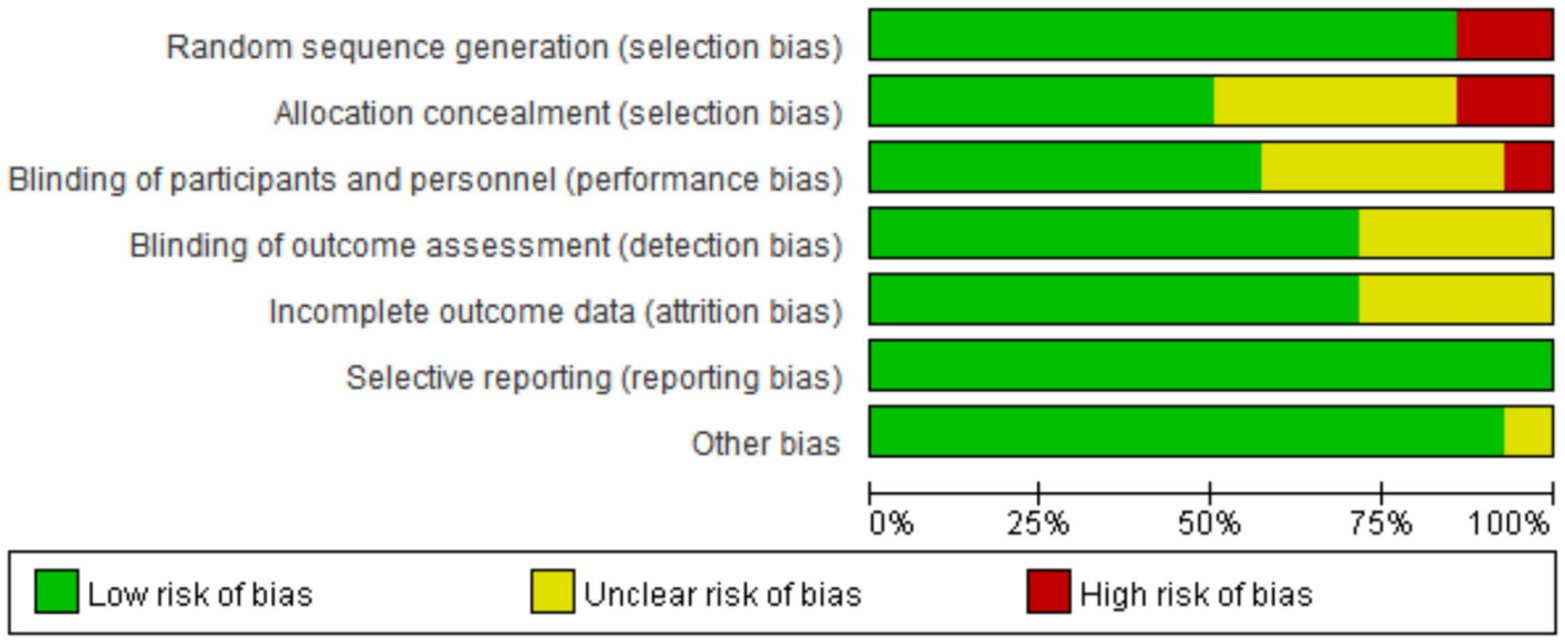
Summary of risk of bias assessment.

**Figure 3:**
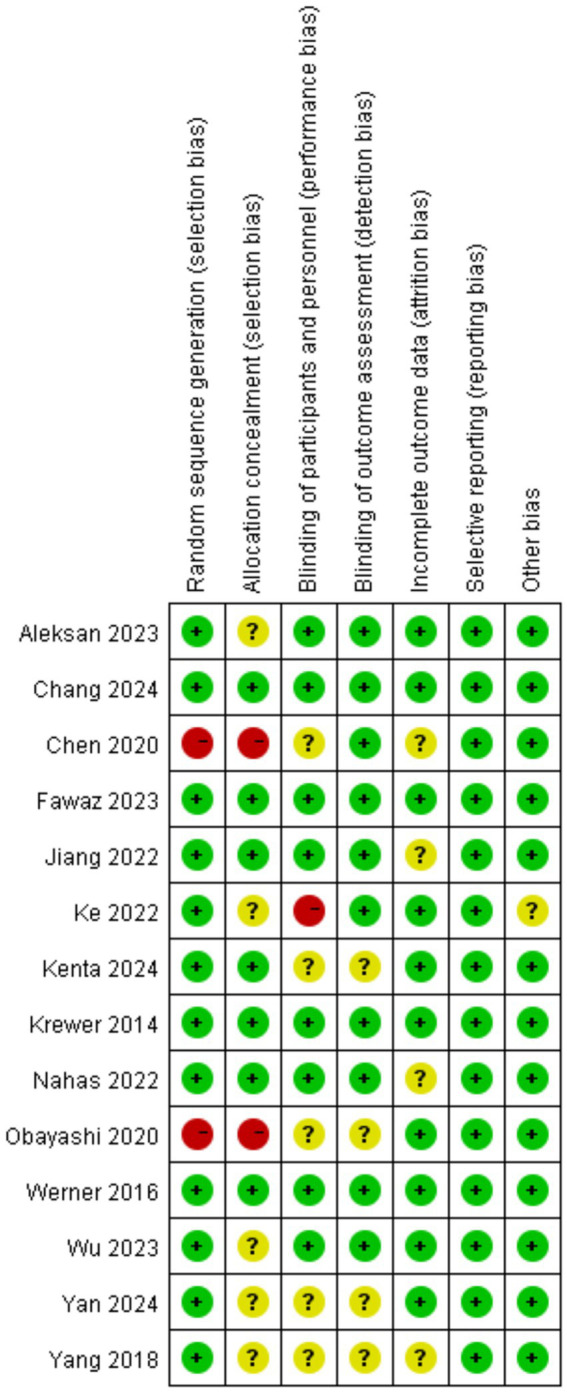
Risk of bias forest plot for included studies.

Evaluation In terms of selection bias, most studies (12/14) employed computer-generated random sequences or drawing lots and were rated as “low risk”; however, Chen et al. (23) and Obayashi and Takahashi (24) were judged as “high risk” due to unspecified randomization protocols. For allocation concealment, 7 studies explicitly described proper methods such as sealed envelopes or independent allocation (20, 21, 25, 27, 28, 31, 33), while the remaining studies lacked detail. Regarding performance bias, over half of the studies utilized sham stimulation, ensuring participant blinding (20, 21, 25, 27–31). For detection bias, 10 studies employed independent third parties to conduct blinded outcome assessments. All included studies presented a low risk of bias concerning incomplete outcome data, selective reporting, and other biases (see Figures 2, 3).

### 3.4 Systematic evaluation and analysis strategy of rPMS

Efficacy using the rPMS parameters from each study, a systematic analysis and progressive stratified effect evaluation were conducted on the post-stroke FMA-UE scores between the experimental and control groups. First, an aggregate analysis of FMA-UE scores across all studies was performed to evaluate the overall clinical effect of rPMS intervention (Section 3.4.1). Given the variability in intervention strategies and stimulation parameters across studies, more detailed subgroup stratified effect evaluations were subsequently conducted based on stroke stage (Section 3.4.2), stimulation frequency (Section 3.4.3), stimulation duration (Section 3.4.4), magnetic density (Section 3.4.5), coil type (Section 3.4.6), and stimulation target (Section 3.4.7).

#### 3.4.1 Overall effect analysis of rPMS on FMA-UE

**Figure 4:**
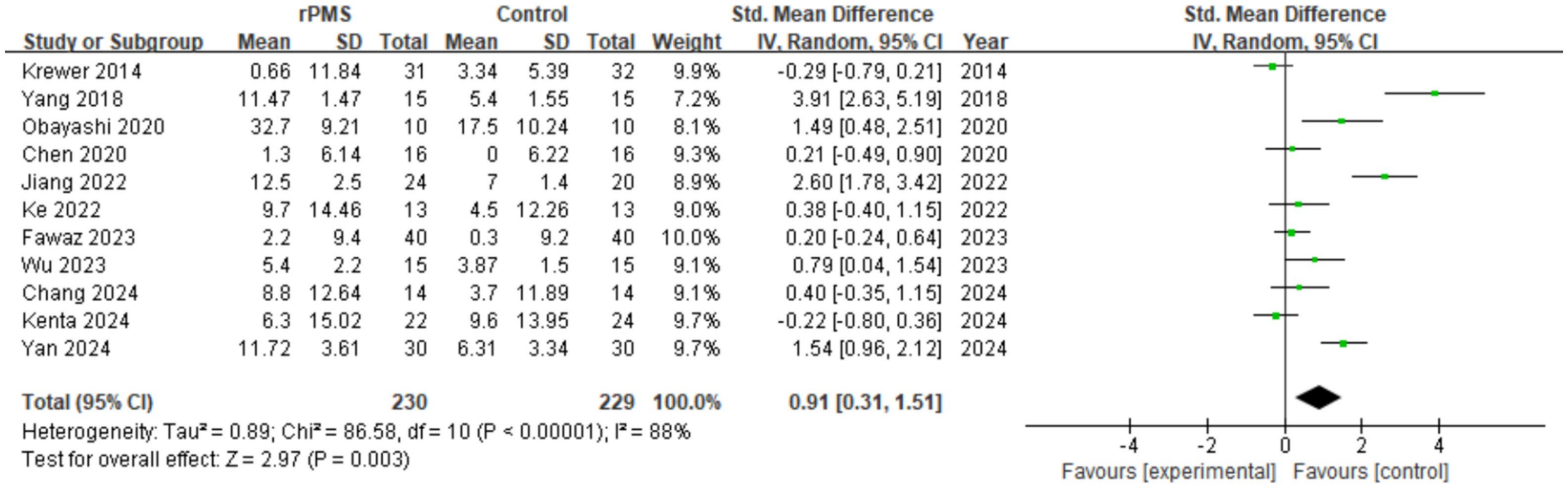
Overall analysis of rPMS on FMA-UE between experimental and control groups.

A total of 11 studies (*n* = 459) were included to assess the difference in FMA-UE scores between the two groups. The results showed that the FMA-UE scores in the rPMS group were significantly superior to those in the control group, with a substantial prognostic effect size (SMD = 0.91, 95% CI 0.31 to 1.51, *p* = 0.003) (see Figure 4). Although the overall effect achieved statistical significance, notable heterogeneity existed among the studies (I^2^ = 88%), necessitating further subgroup stratification or dose-response exploration.

#### 3.4.2 Effect analysis of rPMS on stroke stages

**Figure 5:**
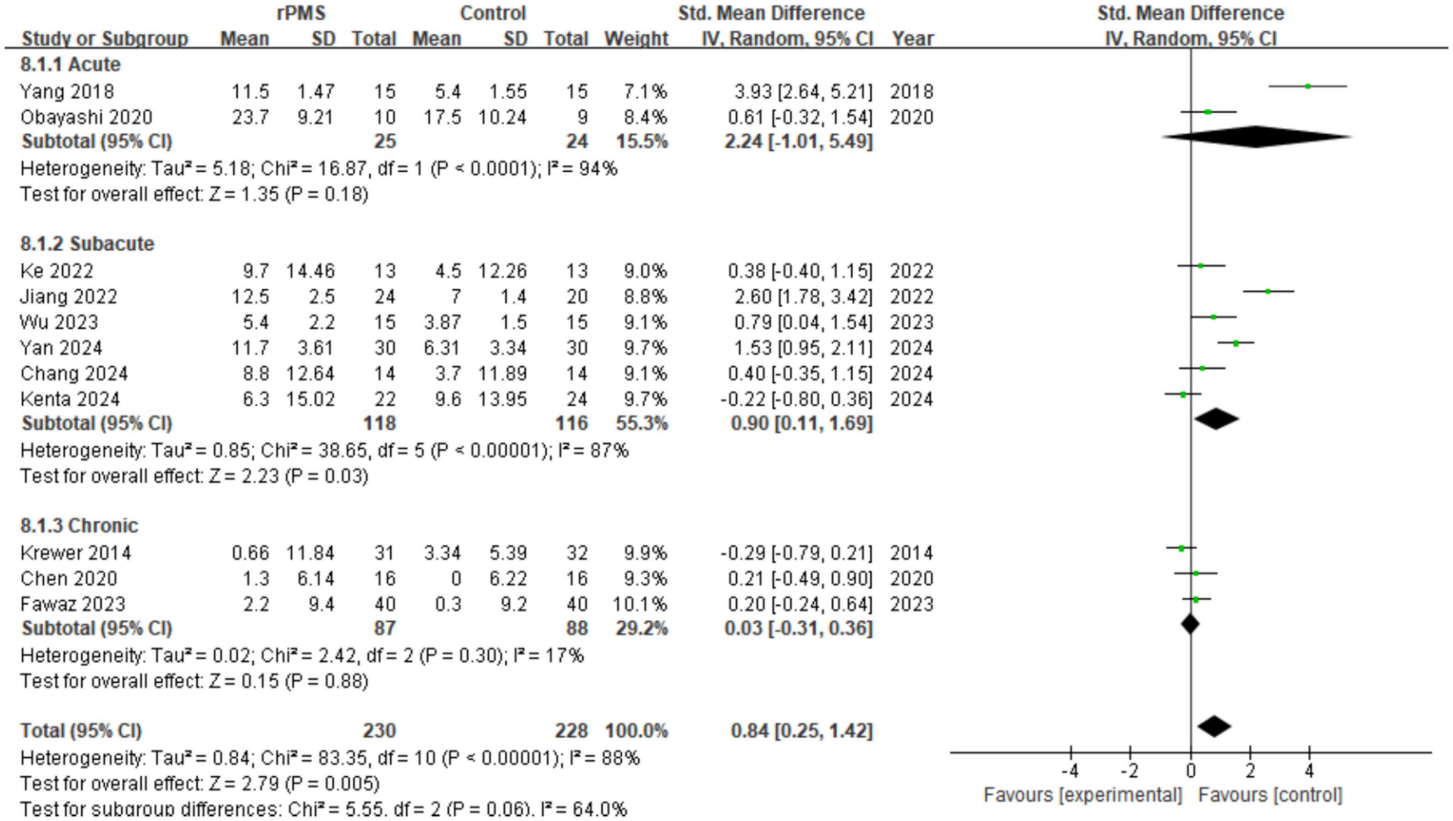
Analysis of rPMS on stroke stages between experimental and control groups.

A total of 11 studies (*n* = 458) were included to evaluate differences in FMA-UE scores across stroke stages. The pooled analysis demonstrated that rPMS exerted a positive effect on score improvement (SMD = 0.84, 95% CI 0.25 to 1.42, *p* = 0.005) (see Figure 5). Specifically, the acute phase (stroke duration <14 days, 2 studies/n = 49) showed a large effect size (SMD = 2.24, 95% CI: −1.01 to 5.49, *p* = 0.18), yet it lacked statistical significance due to wide confidence intervals and was accompanied by extremely high heterogeneity (I^2^ = 94%). This suggests a potential clinical benefit of rPMS in the acute phase, but it must be interpreted cautiously due to sample size limitations and methodological discrepancies (e.g., baseline differences in stroke onset time in Obayashi 2020). In the subacute phase (14 days to 6 months, 6 studies/n = 234), score improvement was significant (SMD = 0.90, 95% CI 0.11 to 1.69, *p* = 0.03), though high heterogeneity remained (I^2^ = 87%). This indicates that the subacute phase is a viable therapeutic window for rPMS intervention. However, in the chronic phase (>6 months, 3 studies/n = 175), the improvement was minimal (SMD = 0.03, 95% CI −0.31 to 0.36, *p* = 0.06), with significantly reduced inter-study heterogeneity (I^2^ = 17%). Findings suggest that rPMS intervention during this late stage provides no significant restorative effect on upper limb motor function. Subgroup tests revealed substantial differences based on staging (*p* = 0.06, I^2^ = 64.0%), highlighting a potential time-dependent characteristic of rPMS efficacy.

#### 3.4.3 Effect analysis of rPMS vs. stimulation frequency (Hz)

**Figure 6:**
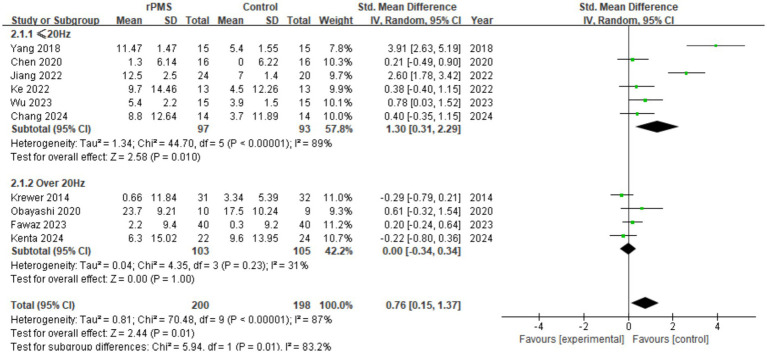
Analysis of rPMS stimulation frequency (Hz) between experimental and control groups.

Ten studies (*n* = 398) were included to assess FMA-UE score differences under varying stimulation frequencies. Pooled analysis revealed that frequency significantly moderated score improvements (SMD = 0.76, 95% CI 0.15 to 1.37, *p* = 0.01) (see Figure 6). Specifically, rPMS efficacy was markedly superior in the ≤20 Hz subgroup (SMD = 1.30, 95% CI 0.31 to 2.29, *p* = 0.010), albeit with high heterogeneity (I^2^ = 89%). Conversely, the >20 Hz subgroup showed no significant efficacy (SMD = 0.00, 95% CI −0.34 to 0.34, *p* = 1.00) with lower heterogeneity (I^2^ = 31%). This indicates that ≤20 Hz is the optimal parameter interval, whereas frequencies >20 Hz render rPMS largely ineffective.

#### 3.4.4 Effect analysis of rPMS vs. stimulation duration

**Figure 7:**
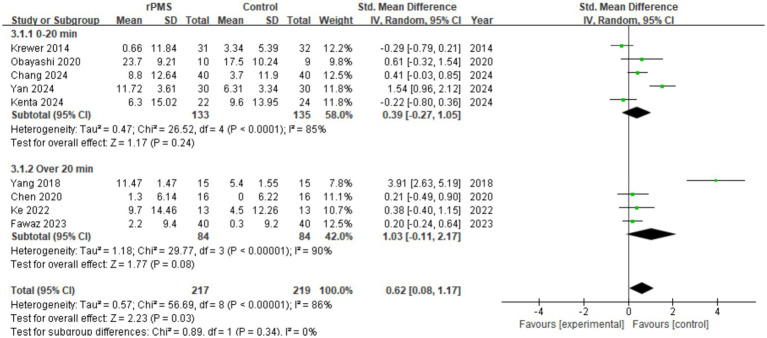
Analysis of rPMS stimulation duration between experimental and control groups.

Nine studies (*n* = 436) evaluated differences in FMA-UE scores based on stimulation duration. The aggregated analysis showed a positive correlation between varying rPMS stimulation times and score changes (SMD = 0.62, 95% CI 0.08 to 1.17, *p* = 0.03) (see Figure 7). The 0–20 min subgroup yielded no statistically significant effect (SMD = 0.39, 95% CI −0.27 to 1.05, *p* = 0.24) with high heterogeneity (I^2^ = 85%). Similarly, the >20 min subgroup lacked significance (SMD = 1.03, 95% CI −0.11 to 2.17, *p* = 0.08) and exhibited high heterogeneity (I^2^ = 90%). Although the overall effect suggests an optimal stimulation duration may exist, confounding variables necessitate further investigation.

#### 3.4.5 Effect analysis of rPMS vs. magnetic density

**Figure 8:**
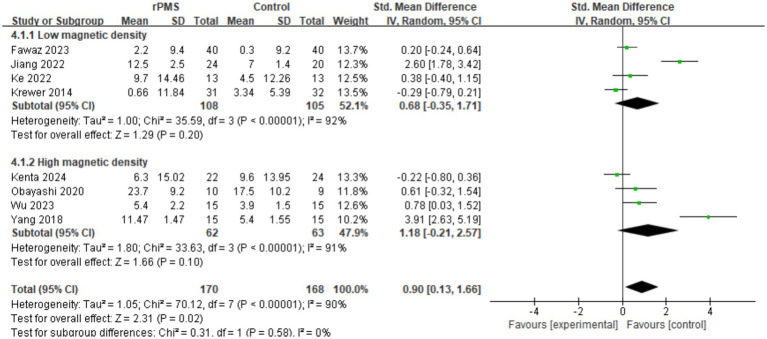
Analysis of rPMS magnetic density between experimental and control groups.

Eight studies (*n* = 388) were analyzed to determine the impact of magnetic density on FMA-UE. The summary analysis demonstrated a positive correlation between magnetic density and clinical improvement (SMD = 0.90, 95% CI 0.13 to 1.66, *p* = 0.02) (see Figure 8). However, stratified analysis revealed no significant correlations within either the low-density subgroup (SMD = 0.68, 95% CI −0.35 to 1.71, *p* = 0.20) or the high-density subgroup (SMD = 1.18, 95% CI −0.21 to 2.57, *p* = 0.10). Given the severe heterogeneity (I^2^ > 90%), larger sample sizes or advanced dose-response analyses are required for definitive conclusions.

#### 3.4.6 Effect analysis of rPMS vs. coil type

**Figure 9:**
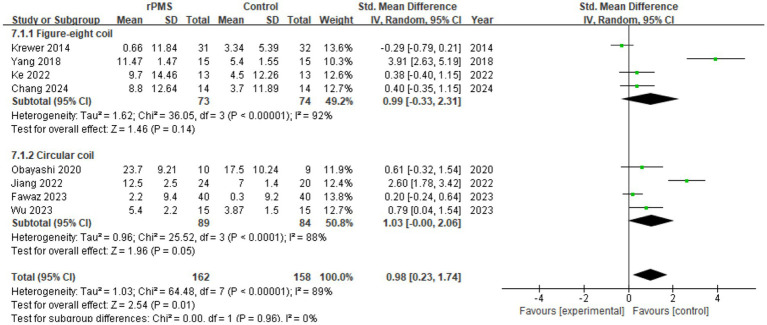
Analysis of rPMS coil types between experimental and control groups.

Eight studies (*n* = 320) assessed the impact of coil types on FMA-UE improvements. The pooled results indicated a significant difference regarding coil variations (SMD = 0.98, 95% CI 0.23 to 1.74, *p* = 0.01) (see Figure 9). Specifically, the figure-eight coil subgroup showed no significant correlation (SMD = 0.99, 95% CI −0.33 to 2.31, *p* = 0.14) with high heterogeneity (I^2^ = 92%). The circular coil subgroup presented a borderline statistical effect (SMD = 1.03, 95% CI 0.00 to 2.06, *p* = 0.05) alongside high heterogeneity (I^2^ = 88%). With no significant inter-group interaction test difference (Chi^2^ = 0.00, *p* = 0.96), current evidence cannot conclusively establish therapeutic disparities between coil designs.

#### 3.4.7 Effect analysis of rPMS vs. target

**Figure 10:**
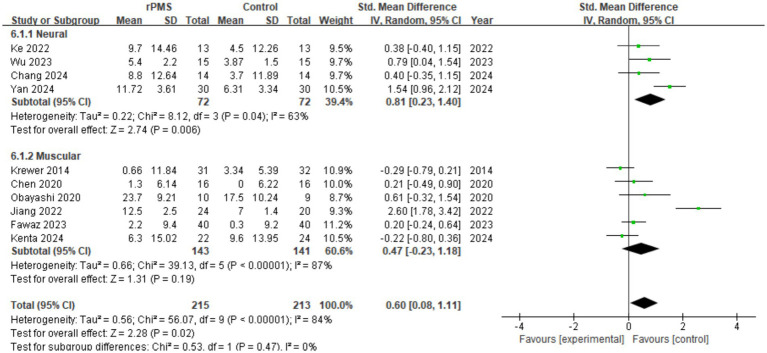
Analysis of rPMS stimulation targets between experimental and control groups.

Ten studies (*n* = 428) were evaluated for FMA-UE differences based on stimulation targets (neural vs. muscular). The aggregated data revealed that target selection significantly influenced score adjustments (SMD = 0.60, 95% CI 0.08 to 1.11, *p* = 0.02) (see Figure 10). Notably, the neural target subgroup achieved a larger effect size (SMD = 0.81, 95% CI 0.23 to 1.40, *p* = 0.006), while the muscular subgroup showed no significant correlation (SMD = 0.47, 95% CI −0.23 to 1.18). Thus, selecting neural targets during rPMS intervention may yield more pronounced clinical benefits.

### 3.5 Effect analysis of rPMS on spasticity (MAS)

**Figure 11:**
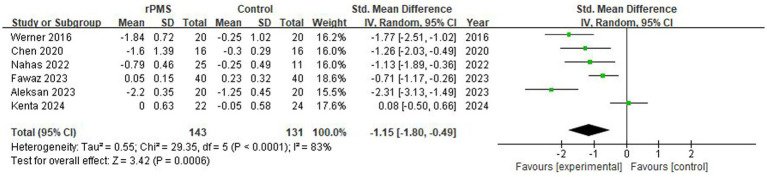
Analysis of MAS degree of rPMS between experimental and control groups.

Six studies (*n* = 274) were included to analyze post-intervention MAS scores. The results confirmed that rPMS intervention substantially alleviated spasticity in post-stroke patients, achieving a large clinical effect size (SMD = −1.15, 95% CI −1.80 to −0.49, *p* = 0.0006) (see Figure 11). Despite strong statistical significance, the high heterogeneity (I^2^ = 83%) and individual variances indicate that confounding parameters still require quantitative dose-response scrutiny.

### 3.6 rPMS dose-response analysis

To investigate the non-linear associations between various rPMS parameters and FMA-UE score changes, researchers employed the REMR model to quantify dose-response relationships, aiming to provide reference guidance for clinical practice. Previous rehabilitation studies have shown that the Minimal Clinically Important Difference (MCID) for post-stroke FMA-UE is 5 to 10 points (34). Reaching this threshold signifies a substantial qualitative leap in the patient’s upper limb motor paradigm (transitioning from flexor synergy to isolated movement).

The REMR results indicated that among rPMS stimulation parameters, frequency (10–20 Hz) and duration (10–20 min) act as strong correlates, exhibiting an inverted U-shaped dose-response curve. Notably, within these optimal stimulation windows, the peak FMA-UE gains (approximately 12.00 to 13.82 points) far exceeded the aforementioned MCID clinical threshold. This confirms that rPMS intervention under specific parameter combinations can facilitate substantial breakthroughs in upper limb motor function for post-stroke patients. Due to standard variations in magnetic field metrics across studies (MSO, MCT, RMT, and Tesla), values were normalized for percentage of maximum stimulator output (% MSO) analysis. The normalization criteria were as follows: 0.65–0.9 Tesla mapped to a 30–45% MSO interval; studies utilizing motor thresholds (RMT/MCT) were converted via relative percentage weighting (e.g., 100% RMT matched the 40–50% MSO range). The normalized results demonstrated that magnetic stimulation intensity (20–55% MSO) and treatment days (14–21 days) served as weak predictors, with rPMS efficacy displaying a plateau or linear trend.

#### 3.6.1 Stimulation frequency

**Figure 12:**
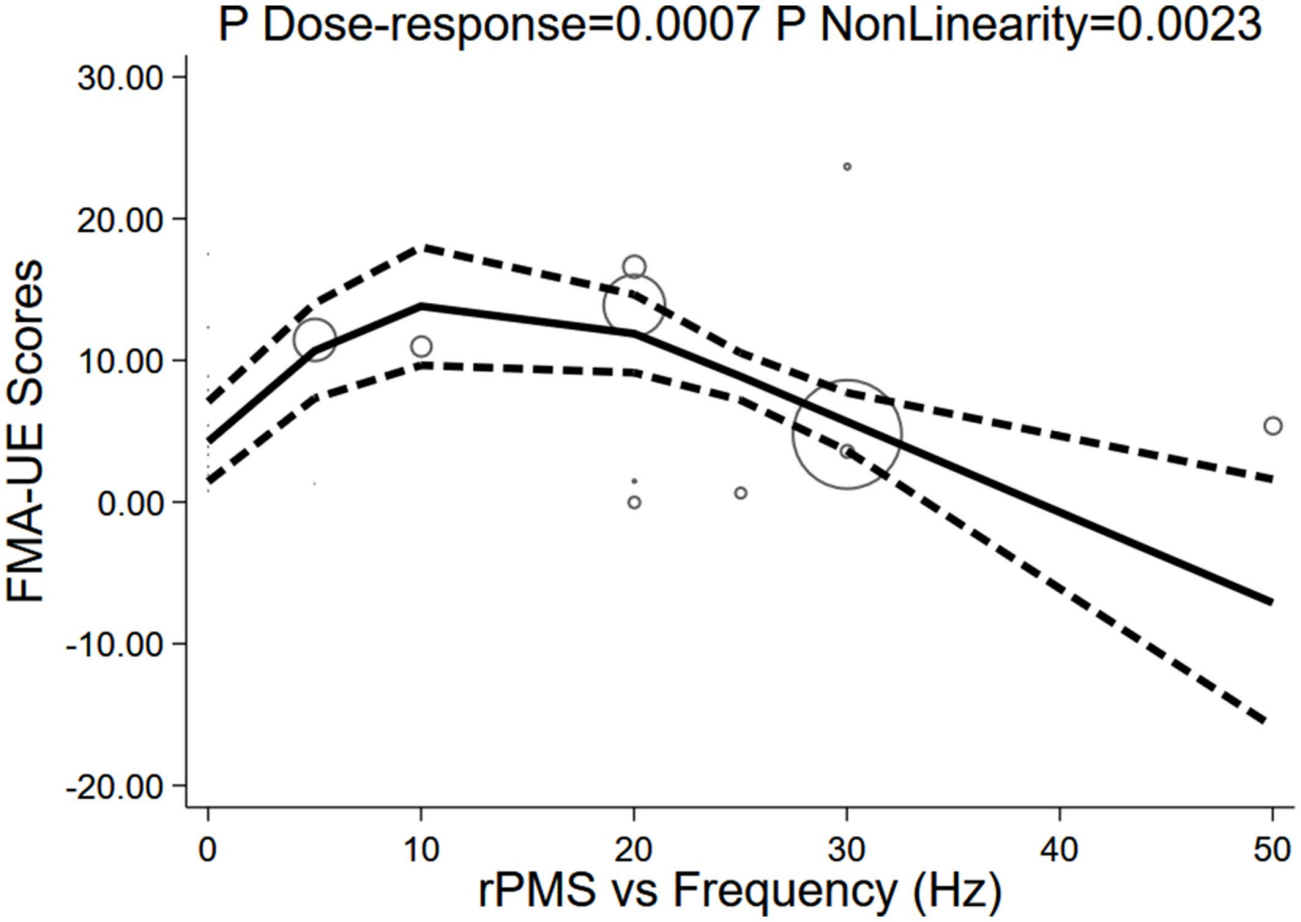
Dose-response of stimulation frequency and FMA-UE.

A significant non-linear relationship was observed between rPMS stimulation frequency and FMA-UE scores (P for dose-response = 0.0007; P for non-linearity < 0.001). The model fit well (R^2^ = 0.554, Root MSE = 2.806). The dose-response curve depicted an inverted U-shape: the baseline (0 Hz) effect was 4.26 points (95% CI: 1.44–7.08), rapidly reaching its peak as frequency increased. The maximum value was attained at 10 Hz (13.82 points, 95% CI: 9.65–18.00), followed by a gradual decline. The effect dropped to 11.89 points (95% CI: 9.13–14.65) at 20 Hz, further decreased to 5.67 points (95% CI: 3.62–7.72) at 30 Hz, and ultimately reversed at 50 Hz (−7.11 points, 95% CI: −15.83 to 1.61). These findings indicate that 10 Hz is the optimal rPMS frequency, with an effective therapeutic window spanning 5–20 Hz (see Figure 12).

#### 3.6.2 Stimulation duration

**Figure 13:**
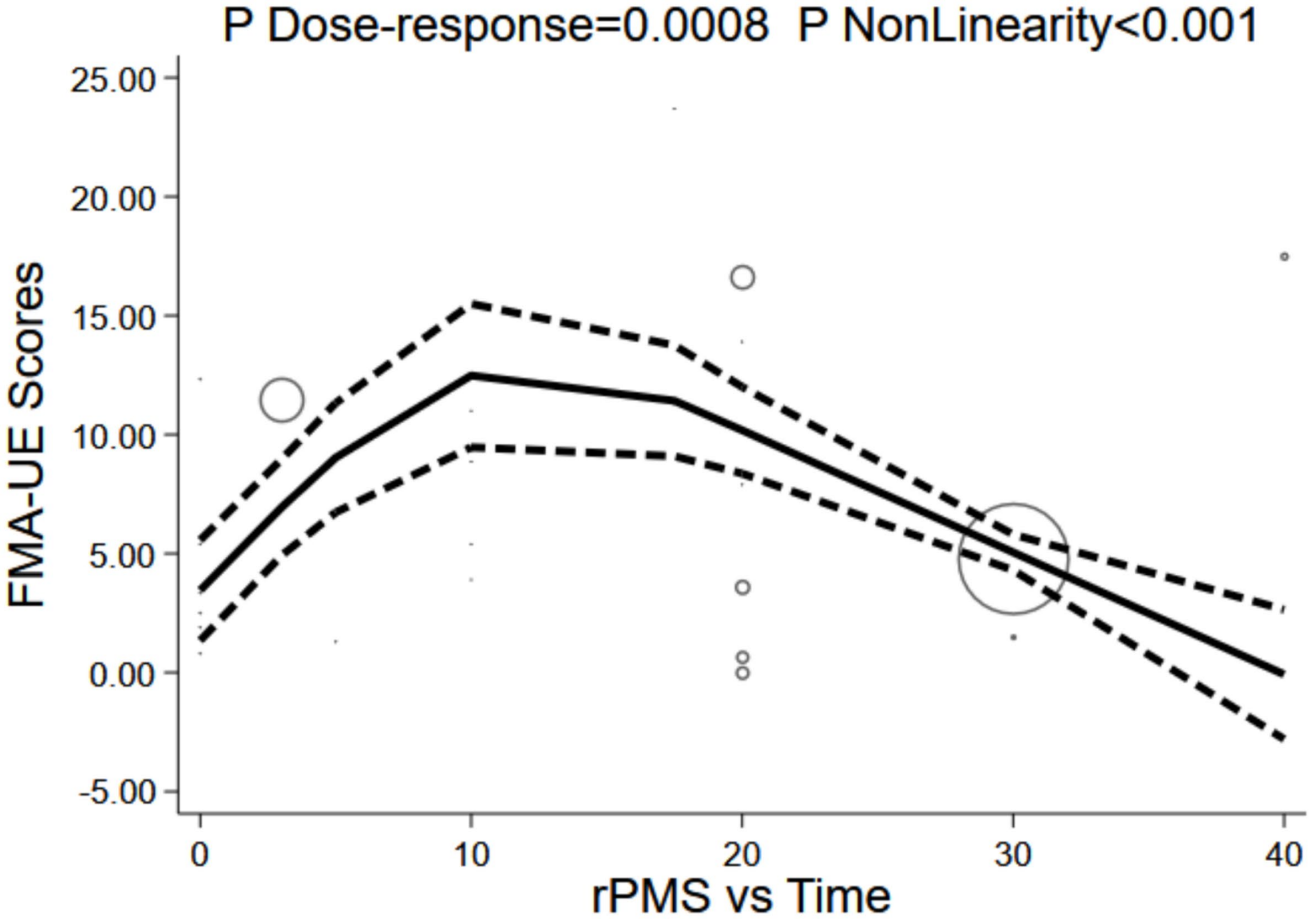
Dose-response of stimulation duration and FMA-UE.

A significant non-linear correlation existed between rPMS stimulation duration and FMA-UE scores (P for dose-response = 0.0008; P for non-linearity < 0.001), showing a robust model fit (R^2^ = 0.535, Root MSE = 2.867). The inverted U-shaped dose-response curve revealed a baseline (0 min) of 3.46 points (95% CI: 1.06–5.86), with score improvements climbing alongside treatment duration until peaking at a plateau at 10 min (gains of roughly 12–13 points), and then trending downward after 20 min. A duration of 10–20 min maximizes proprioceptive sensory input and cortical plasticity induction, whereas exceeding 30 min may trigger neural homeostatic inhibition or synaptic efficacy saturation (see Figure 13).

#### 3.6.3 Magnetic density

**Figure 14:**
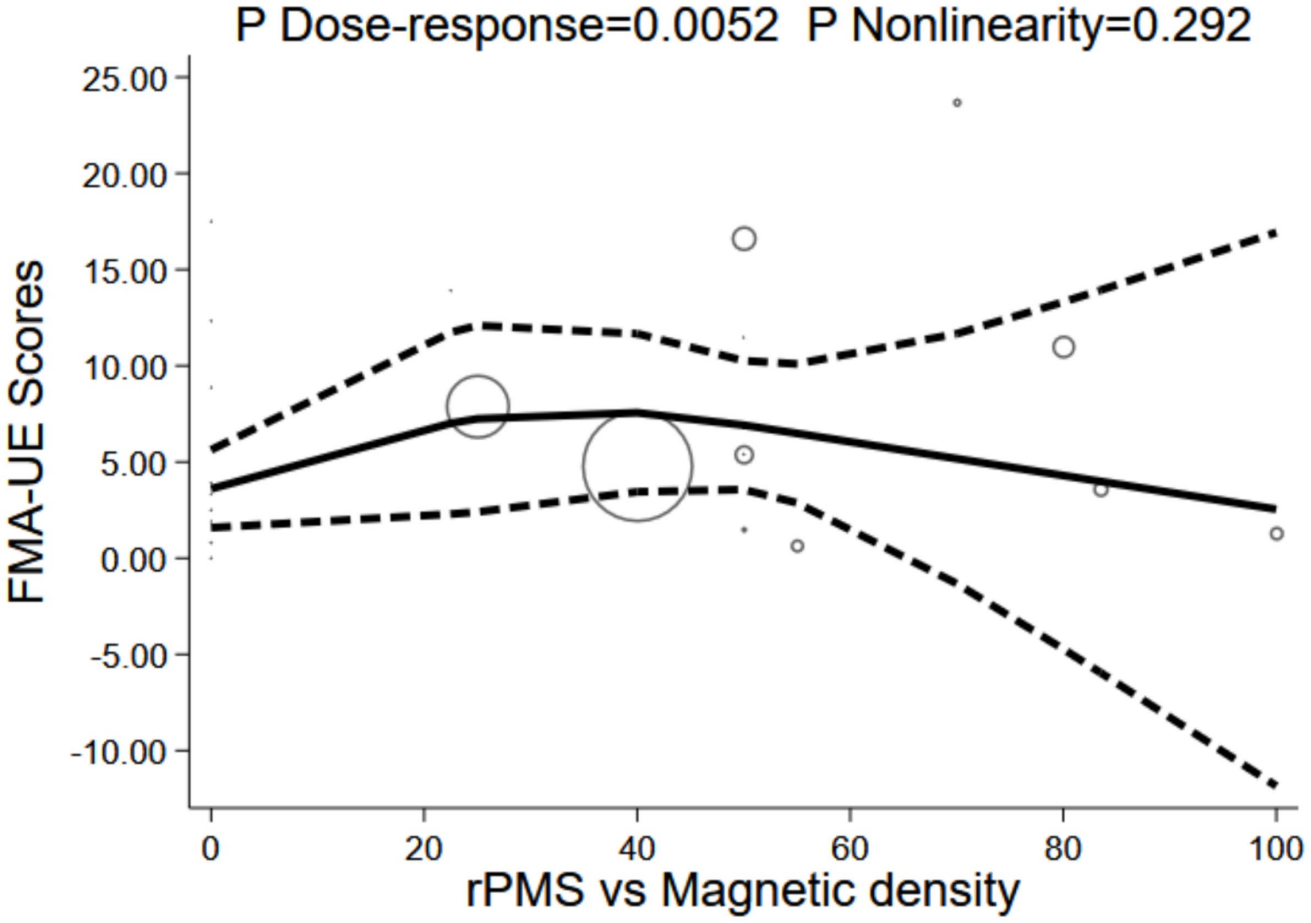
Dose-response of magnetic density and FMA-UE.

Magnetic stimulation intensity displayed a significant non-linear relationship with FMA-UE scores (P for dose-response = 0.0052), although the test for non-linearity was non-significant (P for non-linearity = 0.292), and the model fit was limited (R^2^ = 0.211, Root MSE = 3.734). The dose-response curve exhibited a mild inverted U-shape: the baseline (0%) stood at 3.61 points (95% CI: 1.59–5.63), rising slowly with increased intensity to reach a peak plateau at 40% (7.56 points, 95% CI: 3.45–11.67), before gradually attenuating and dropping to 2.54 points (95% CI: −11.84 to 16.93) at 100% intensity. The effect size remained relatively stable (6.48–7.24 points) across the peak interval (22.5–55%); however, the high-dose region (>70%) generated extremely wide confidence intervals with lower bounds turning negative. This implies that low-to-moderate intensity (20–55%) serves as the optimal window; high-intensity protocols offer no incremental benefit and might dilute the therapeutic effect due to coil overheating or unwanted spread of the stimulation area. Hence, magnetic intensity should be calibrated within the 20–55% MSO range rather than pushing toward maximum output thresholds (see Figure 14).

#### 3.6.4 Treatment days

**Figure 15:**
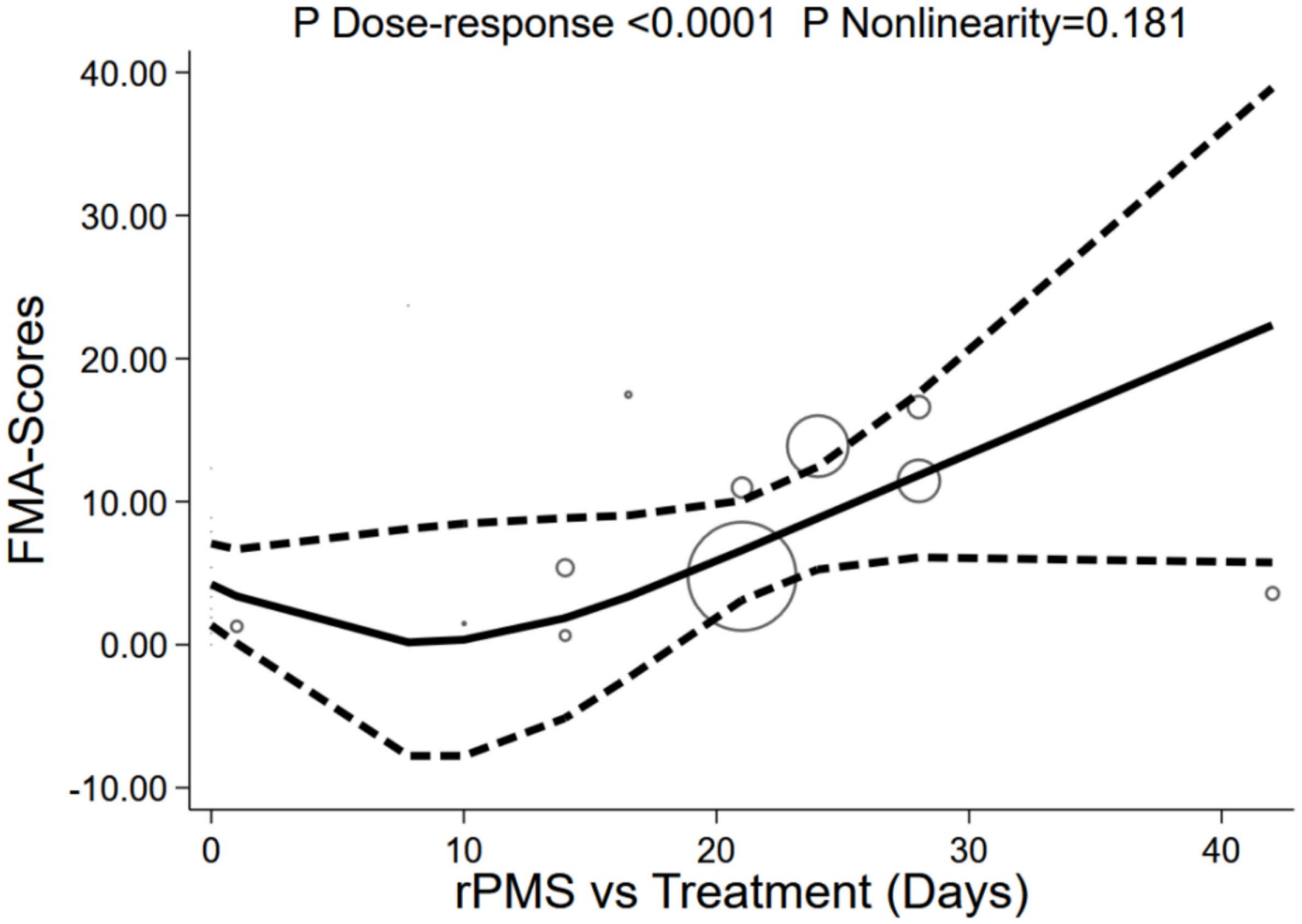
Dose-response of treatment days and FMA-UE.

The number of rPMS treatment days significantly correlated with FMA-UE scores, showcasing a notable dose-dependent growth trend (P for dose-response < 0.0001), despite a non-significant non-linearity test (P for non-linearity = 0.181) and moderate model fit (R^2^ = 0.377, Root MSE = 3.318). The dose-response curve demonstrated that FMA-UE scores initially plateaued before rising continuously as treatment days extended: the baseline effect was 4.22 points (95% CI: 1.36–7.07), entering a plateau phase at 10 days (0.35 points), surging rapidly to 6.59 points (95% CI: 3.10–10.07) at 21 days, and ultimately peaking at 22.34 points on day 42. Although the confidence interval at day 42 was wide (95% CI: 5.75–38.92), the overall trend of score improvement remained stable. Current evidence suggests that the duration of treatment days acts as a key driver for FMA-UE improvements, with particularly pronounced rehabilitation benefits unlocked when extending treatment regimens to ≥21 days (see Figure 15).

## 4 Discussion

Through meta-analysis and dose-response regression models, this study systematically quantified the “optimal intervention window” and “optimal stimulation parameters” of rPMS for post-stroke upper limb motor recovery. The findings indicate that rPMS facilitates the restoration of motor dysfunction and significantly mitigates muscle spasticity caused by stroke. These benefits are particularly prominent when applied during the subacute phase utilizing low frequency (≤20 Hz), low-to-moderate magnetic intensity (20–55% MSO), neural target stimulation, and an extended treatment course (≥21 days). In fact, rather than serving as a mere muscle-strengthening exercise, rPMS operates as a neuroplasticity modulator built upon a “peripheral-central closed-loop circuit” (35). By inducing high-intensity proprioceptive afferents, rPMS activates Group Ia and II sensory fibers that project upwards via the dorsal root ganglion to the primary sensory cortex, eventually modulating the excitability of the primary motor cortex (M1) through U-fibers (36). Research by Maeda et al. demonstrates that this “sensory-driven motor” paradigm can induce plastic alterations within M1, ultimately reconnecting damaged corticospinal tracts (37). Benefiting from its superior penetrative capacity and adaptability, rPMS successfully recruits deep mechanoreceptors such as muscle spindles and Golgi tendon organs, generating afferent signals that more closely mimic physiological patterns. This proves especially advantageous for restoring fine motor skills when compared to conventional physical therapy (38).

This study identified that rPMS achieves optimal efficacy at stimulation frequencies of ≤20 Hz, whereas high-frequency stimulation fails to yield additional benefits. This phenomenon is likely tethered to the “bio-resonance” of the brain’s intrinsic oscillatory frequencies. Notably, the 10–20 Hz range perfectly corresponds to the beta band of the sensorimotor cortex, a frequency domain strictly correlated with motor recruitment status and corticomuscular coherence (39, 40). Furthermore, research by Flood et al. highlights that excessively high frequencies (e.g., >25 Hz) trigger sensory gating mechanisms, prompting the central nervous system to suppress or dampen incoming afferent signals and down-regulate cortical hyperactivity (41). Thus, 20 Hz functions as the “sweet-spot frequency” for rPMS, elegantly balancing peripheral activation and central reception. Regarding the threshold effect of magnetic intensity, the dose-response curve suggests that the 20–55% MSO low-to-moderate range provides ample stimulation to yield significant therapeutic effects, whereas intensities pushed too high precipitate a plateau or even a decline in efficacy. This phenomenon likely implicates mechanisms of “homeostatic plasticity”: excessively intense peripheral sensory inputs provoke a protective synaptic down-regulation, effectively blocking neural network responsiveness (42). Moreover, high-intensity stimuli can easily trigger antagonist muscle co-contraction or nociceptive withdrawal reflexes, thereby inhibiting plastic muscular adaptations. While an enormous effect size was recorded during the acute stroke phase (SMD = 2.24), the restricted sample size (*n* = 49) spawned overly wide confidence intervals and high heterogeneity, thereby impacting the statistical power and robustness of the evidence. In contrast, the subacute phase presented statistically stable parameters (*n* = 234) and delivered much stronger evidence backing its status as the “optimal therapeutic window” for rPMS. This finding perfectly aligns with the “critical period” theory of post-stroke neural repair. During this highly active subacute phase characterized by up-regulated brain-derived neurotrophic factor expression and vigorous synaptic reorganization, rPMS efficiently guides functional circuit reconstruction (43). Theoretically, neural plasticity mechanisms are severed when stroke damages the corticospinal or sensory tracts. Yet, despite the confounding variables of different stroke subtypes within this study, the therapeutic impact of rPMS remained robust (SMD = 0.91). This suggests that rPMS inherently possesses central remodeling capacities while retaining the ability to transmit signals to the cortex even when pathways are compromised, thereby passively amplifying motor unit recruitment in targeted muscles, independently inducing neural network remodeling, and initiating rhythmic muscle contractions. Conversely, chronic-phase patients have typically developed maladaptive compensatory movements or secondary soft-tissue adhesions intricately linked to severe damage in the corticospinal or sensory pathways. Given the mechanism of action of rPMS, if a massive brain lesion entirely blocks these pathways, the “peripheral-central-peripheral” closed-loop system is rendered dysfunctional, making it tremendously difficult to alter the established pathological homeostasis solely through rPMS (44, 45). When determining stimulation targets, applying rPMS to nerve roots (e.g., brachial plexus, radial nerve) demonstrated superior efficacy over targeting muscle bellies. Nerve root stimulation synchronously recruits a vastly broader range of motor units and sensory fibers, accelerates afferent neural progression, and sparks widespread desynchronized activation across the cerebral cortex (46).

The pronounced capability of rPMS to reverse spasticity (SMD = −1.15) suggests its mechanism extends beyond cortical modulation to actively suppressing hyperactive spinal reflexes. By inducing rhythmic cycles of muscle contraction and relaxation, rPMS triggers presynaptic inhibitory mechanisms that subsequently temper the hyperactive stretch reflex arc. This is of immense clinical value in dismantling the vicious cycle of “spasticity-immobilization-exacerbated spasticity” (47, 48). The analyses within this study confirmed that rPMS holds dual characteristics of “immediacy” and “after-effects” in relieving post-stroke spasticity across both short-term (1–2 days) and mid-term (14–21 days) studies. However, due to a distinct lack of long-term follow-up data spanning several months and the absence of independent stratification across stroke subtypes within the included literature, a comprehensive multidimensional assessment evaluating the full risk–benefit ratio remains unattainable; specifically, it remains uncertain whether rPMS can induce permanent, anti-spastic remodeling within neural networks.

In the long run, the FMA-UE score enhancements and spasticity alleviation catalyzed by rPMS deliver tremendous functional benefits to patients battling post-stroke motor deficits. Breaking past the MCID threshold for FMA-UE scores signifies the clinical correction of abnormal flexor synergy patterns and the re-establishment of isolated, voluntary shoulder and elbow movements (49). These isolated movements serve as the critical biomechanical foundation necessary for “reaching” and “goal-directed grasping” (50). Building upon this, the recruitment mechanisms elicited by rPMS in muscle tissues effectively upgrade the fine motor control of the affected limb, directly supporting patient independence in crucial activities of daily living (ADL) such as eating and dressing, and significantly alleviating caregiver burdens (51). Furthermore, substantial spasticity mitigation inherently lowers the risk of severe joint and muscle contractures, effectively releasing the passive range of motion within the afflicted limb (52). Consequently, rPMS does far more than physiologically soothe the discomfort of stroke patients—it actively empowers their functional reintegration into daily life.

Additionally, while dose-response analyses have revealed statistically significant correlations between stimulation parameters and motor improvements, the exact biological mechanisms remain firmly in the realm of hypothesis. Future clinical trials must integrate multimodal neuroimaging tools such as fMRI, TMS-EEG, or functional Near-Infrared Spectroscopy (fNIRS) to meticulously validate the spatiotemporal dependencies between rPMS dosages, cortical excitability, and neural plasticity, thereby transforming statistical trends into direct neurophysiological evidence (53).

## 5 Conclusion

By systematically analyzing the dose-response relationship between rPMS parameters and post-stroke FMA scores, this study established that applying rPMS during the subacute phase utilizing low frequency (≤20 Hz), low-to-moderate intensity (20–55% MSO), neural-targeted stimulation, and an extended course (≥21 days) represents the optimal parameter framework. rPMS exerts a highly significant modulatory effect on beta-band corticomuscular coherence and homeostatic plasticity mechanisms. Furthermore, the efficacy of rPMS in ameliorating post-stroke spasticity demonstrated a high degree of consistency. In summary, the findings of this study unveil crucial stimulation parameters, thereby offering novel scientific evidence and clinical recommendations for the implementation of rPMS in post-stroke rehabilitation.

## Data Availability

The original contributions presented in the study are included in the article/Supplementary material, further inquiries can be directed to the corresponding author.
